# GPR92 activation in islet macrophages controls β cell function in a diet-induced obesity model

**DOI:** 10.1172/JCI160097

**Published:** 2022-11-01

**Authors:** Camila O. de Souza, Vivian A. Paschoal, Xuenan Sun, Lavanya Vishvanath, Qianbin Zhang, Mengle Shao, Toshiharu Onodera, Shiuhwei Chen, Nolwenn Joffin, Lorena M.A. Bueno, Rana K. Gupta, Da Young Oh

**Affiliations:** 1 Touchstone Diabetes Center, Department of Internal Medicine, University of Texas Southwestern Medical Center, Dallas, Texas, USA.; 2 Division of Endocrinology, Department of Medicine, Duke Molecular Physiology Institute, Durham, North Carolina, USA.; 3 The Center for Microbes, Development and Health, Institute Pasteur of Shanghai, Chinese Academy of Sciences, Shanghai, China.

**Keywords:** Inflammation, Metabolism, Diabetes, G protein&ndash;coupled receptors, Islet cells

## Abstract

The molecular mechanisms underlying obesity-induced increases in β cell mass and the resulting β cell dysfunction need to be elucidated further. Our study revealed that GPR92, expressed in islet macrophages, is modulated by dietary interventions in metabolic tissues. Therefore, we aimed to define the role of GPR92 in islet inflammation by using a high-fat diet–induced (HFD-induced) obese mouse model. GPR92-KO mice exhibited glucose intolerance and reduced insulin levels — despite the enlarged pancreatic islets — as well as increased islet macrophage content and inflammation level compared with WT mice. These results indicate that the lack of GPR92 in islet macrophages can cause β cell dysfunction, leading to disrupted glucose homeostasis. Alternatively, stimulation with the GPR92 agonist farnesyl pyrophosphate results in the inhibition of HFD-induced islet inflammation and increased insulin secretion in WT mice, but not in GPR92-KO mice. Thus, our study suggests that GPR92 can be a potential target to alleviate β cell dysfunction via the inhibition of islet inflammation associated with the progression of diabetes.

## Graphical abstract

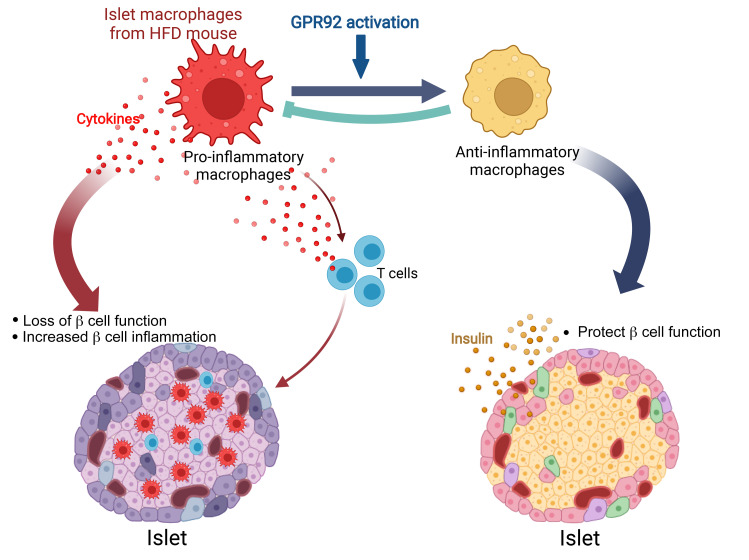

## Introduction

Obesity-induced low-grade tissue inflammation manifests in multiple tissues, including pancreatic islets, and plays a critical role in mediating insulin resistance and β cell dysfunction (1–3). Obesity-associated insulin resistance stimulates a compensatory hypertrophy of the islets of Langerhans by increasing the β cell mass. This increase in β cell mass leads to a transient period of hyperinsulinemia and eventually causes β cell failure, thereby reducing insulin secretion (4–6). The underlying molecular mechanisms that trigger this compensatory increase in β cell mass and its subsequent demise remain elusive. However, as inflammation is the most common feature observed in the pancreatic islets of patients who are obese with type 2 diabetes (T2D) (3, 7–9), it can be hypothesized that islet inflammation is pivotal in inducing β cell dysfunctions.

During the course of obesity development, pancreatic islets exhibit a progressive accumulation of immune cells (10–12), and consequently high levels of inflammatory cytokines and chemokines (1, 7, 9, 12). Islet inflammation and β cell dysfunction in obesity are intricately associated with islet macrophage (IM) expansion (8, 10–12). IMs detect β cell activity and induce the activation and recruitment of proinflammatory macrophages (M1 macrophages), thereby orchestrating initial inflammatory responses (13). Additionally, studies have challenged the stereotypical perspective that T2D is solely a metabolic disease and identified an autoimmunity component of T2D that overlaps with type 1 diabetes (T1D), i.e., increased β cell apoptosis triggered by the activation of T cells (CD8^+^ and CD4^+^) (14–16).

T cells and macrophages contribute to the rapid increase in islet inflammation by further releasing inflammatory cytokines. Therefore, they contribute to the persistence of inflammatory reactions within the pancreas and worsen autoimmune β cell depletion (8, 9, 11, 16). These proof-of-concept studies indicate the crucial role of immune cell recruitment and islet inflammation in the pathogenesis of T2D, highlighting the potential advantages of targeting inflammatory mediators as an effective treatment strategy for this disease.

G protein–coupled receptors (GPCRs) are the most prevalent signal-transducing proteins on the cell surface. GPCRs regulate numerous physiological and pathological processes, making them the most promising and crucial targets for novel drug discovery and effective therapeutic strategies (17). We previously identified farnesyl pyrophosphate (FPP) as the most potent ligand for GPR92 stimulation. Lysophosphatidic acid (LPA) also induces GPR92 activation; therefore, GPR92 is also called LPAR5 (18).

Since the identification of GPR92 agonists, several physiological functions of GPR92 have been elucidated. GPR92 plays an important role in nociception and pain hypersensitivity (19, 20). GPR92-KO mice are highly susceptible to neuropathic pain (20, 21). Moreover, GPR92 is known to be associated with cell mobility and cancer progression, as evidenced by the fact that GPR92-KO sarcoma cells exhibit increased cell motility, which diminishes when GPR92 is overexpressed (22). Nevertheless, only a few studies have investigated the association of GPR92 with immune functions (23, 24), and there is a significant dearth of knowledge regarding the effects of GPR92 on pancreas metabolism. Here, we aimed to define the role of GPR92 in islet inflammation using a diet-induced mouse model of obesity.

## Results

### GPR92 was highly expressed in IMs.

Our data suggest that GPR92 might play an important role in metabolism. Among the several GPCRs surveyed by RNA-Seq, GPR92 was modulated by dietary interventions in metabolic tissues (GSE198012; ref. 25).

**Figure 1:**
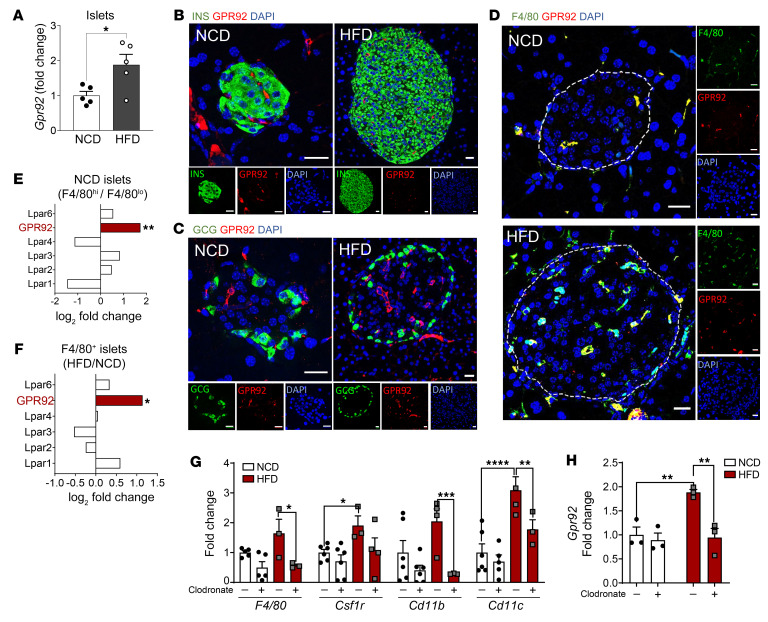
GPR92 is highly expressed in islet macrophages. (**A**) *Gpr92* mRNA expression in the islets of WT mice on a NCD versus a HFD. *n* = 5/group. (**B**–**D**) Immunofluorescence of islets from mice fed with a NCD (original magnification, ×40) or a HFD (original magnification, ×20). INS, insulin; GCG, glucagon. Scale bars: 20 μm. (**E**) Differential expression level of GPR92(LPAR5) versus others LPARs in F4/80^hi^ IM of WT-NCD mice by RNA-Seq data analysis (GSE112002). (**F**) Differential expression level of GPR92(LPAR5) versus others LPARs in F4/80^+^ cells from islets of WT mice on a HFD versus a NCD by RNA-Seq data analysis (GSE112002). (**G**) Gene expression of macrophage markers, *F4/80*, *Csfr1r*, *Cd11b*, *Cd11c* in the islets of WT-HFD mice cultured in vitro with clodronate (+) or control liposome (–) (7 mg/mL) for 24 hours, *n =*3/group. (**H**) Gene expression of *Gpr92* in the islets cultured as indicated in **G**, *n =* 3/group. See Supplemental Table 1 for primers sequences. Fold change normalized by *Rpl19* expression of (**A**) NCD or (**G** and **H**) NCD liposome (–). Data and images are representative of at least 3 independent experiments. All data are expressed as mean ± SEM. ****P < 0.0001, ***P < 0.001, **P < 0.01, *P < 0.05 by 2-way ANOVA with Bonferroni’s post hoc.

Here, we showed that GPR92 was abundantly expressed in the murine bone marrow, pancreas, several GI organs (stomach, small intestine, and colon), skeletal muscle, and dorsal root ganglion (18); Supplemental Figure 1A; supplemental material available online with this article; https://doi.org/10.1172/JCI160097DS1). Interestingly, pancreatic islets (Figure 1A) and stromal vascular fraction from white adipose tissue (Supplemental Figure 1B) of obese, high-fat diet–fed (HFD-fed) mice exhibited an increased expression of GPR92 compared with that in lean, normal chow diet–fed (NCD-fed) mice. Immunofluorescence of islets from mice fed with NCD or HFD revealed that GPR92 was not expressed in cells producing insulin, such as β cells (Figure 1B) or glucagon, such as α cells (Figure 1C). In addition, F4/80^+^ cell counts were considerably increased in the islets from HFD-fed mice, and GPR92 colocalized with the macrophage marker F4/80 (Figure 1D).

Although the role of GPCRs in adipose tissue macrophages in obese mouse models has been extensively studied, the role of immune cells in other metabolic tissues such as pancreas is less appreciated. From analysis of previously deposited transcriptome data of IMs by Ying et al. (8), we confirmed that GPR92 is mostly upregulated in F4/80^+^ cells (Figure 1E) and in the IMs of HFD-fed obese mice (Figure 1F). Additionally, GPR92 was highly expressed in FACS-sorted F4/80^+^CD11b^+^ cells (IMs), but not in F4/80^–^CD11b^–^ cells from isolated islets, while insulin mRNA was highly enriched in F4/80^–^CD11b^–^ cells (Supplemental Figure 1D). These results indicate that GPR92 is not expressed in the endocrine cells of pancreatic islets, but predominantly in the F4/80^+^ immune cell population, which is increased during obesity.

As GPR92 was found to be expressed in these immune cells, we investigated GPR92 expression in the immune cells of other tissues. We observed that GPR92 expression was unaffected by HFD in peritoneal macrophages (pMacs) or microglia. However, HFD significantly increased GPR92 mRNA expression in IMs (Supplemental Figure 1C; ref.^26^).

Next, to investigate the intricate correlation between IMs and GPR92, we depleted IMs by treating them with clodronate. The expression of macrophage markers *F4/80*, *Cd11b*, and *Cd11c*, which were enhanced by a HFD, were downregulated by clodronate treatment (Figure 1G), clearly indicating a successful IM depletion. Similar to the macrophage markers, HFD feeding enhanced *Gpr92* expression in islets, and clodronate substantially reduced *Gpr92* expression levels (Figure 1H).

### Lack of GPR92 expression led to glucose intolerance via reduced insulin secretion.

Obesity-induced islet inflammation can locally abrogate the islet endocrine cell function and significantly reduce insulin secretion by β cells (1, 3, 8). As *Gpr92* was overexpressed in and colocalized with IMs (Figure 1D), we hypothesized that GPR92 depletion might impair the metabolic function of islets.

**Figure 2:**
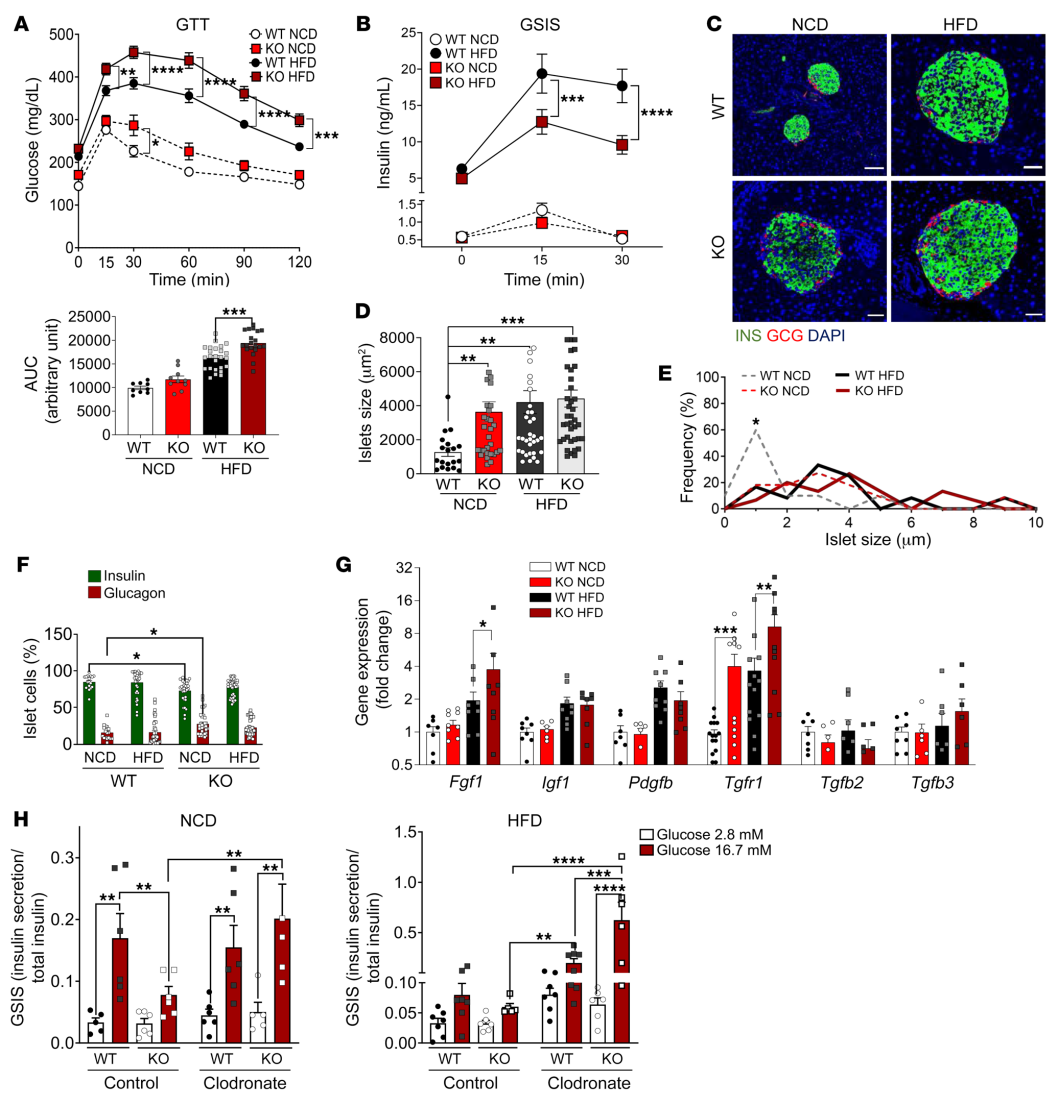
Lack of GPR92 expression leads to glucose intolerance via reduced insulin secretion induced by IMs. (**A**) GTT in WT or KO mice on a NCD or a HFD and their respective AUCs. Glucose 1.5 g/kg injected i.p., *n =* 9–20/group. (**B**) GSIS in WT or KO mice on a NCD or a HFD. Oral gavage of glucose at 1.5 g/kg, *n =* 12–23/group. (**C**) Immunofluorescence of the pancreas. Insulin (green), glucagon (red) and DAPI (blue). Scale bars: 20 μm. (**D**) Islet size (μm^2^) and (**E**) histogram of frequency of islets per size, calculated from IF stained islets in C, *n =* 20–40 islets from 5 mice/group. (**F**) Percentage of insulin^+^ and glucagon^+^ cells in islets IF, *n =* 20–40 islets/group. (**G**) Gene expression of growth factors in islets, *n =* 3–5/group. (**H**) GSIS of the islets of WT or KO mice on a NCD (left panel) or a HFD (right panel) cultured in vitro with clodronate or control liposome (7 mg/mL) for 24 hours, *n =* 5–8/group. See Supplemental Table 1 for primer sequences. Fold change normalized by *Rpl19* expression of WT-NCD. Data and images are representative of at least 3 independent experiments. All data are expressed as mean ± SEM. ****P < 0.0001, ***P < 0.001, **P < 0.01, *P < 0.05 by 2-way ANOVA with Bonferroni’s post hoc.

To this end, we evaluated the effect of NCD and HFD on the metabolic phenotype of GPR92-KO mice compared with WT mice. We observed that HFD-fed GPR92-KO mice exhibited severe glucose intolerance (Figure 2A).

Interestingly, despite having a slightly higher body weight (Supplemental Figure 2A), GPR92-KO mice fed with a HFD were not more insulin resistant than obese WT mice (Supplemental Figure 2B). The white adipose tissue from HFD-fed GPR92-KO mice did not exhibit alterations in size of adipocytes (Supplemental Figure 2, C and D or macrophage infiltration (Supplemental Figure 2, D and E). The elevated glucose level in HFD-fed GPR92-KO mice was caused by decreased insulin (Figure 2B) and C-peptide secretion (Supplemental Figure 2F). Nevertheless, the mRNA expression of the insulin counterregulatory hormone glucagon was upregulated in the islets of HFD-fed GPR92-KO mice compared with WT mice under identical experimental conditions (Supplemental Figure 2G).

In addition to these metabolic aberrations, the islets from GPR92-KO mice were strikingly larger compared with WT mice, particularly with a NCD (Figure 2, C–E), and exhibited a higher proportion of glucagon^+^ cells and lower insulin^+^ cells (Figure 2, C and F). Concurrently, KO islets exhibited a remarkably higher expression of growth factors (Figure 2G). The islet size in NCD-fed GPR92-KO mice was comparable to that of the HFD-fed WT and GPR92-KO mice (Figures 2, C–E). However, NCD-fed GPR92-KO mice were only marginally glucose-intolerant (Figure 2A) and did not exhibit insulin resistance (Supplemental Figure 2B) or irregular insulin secretion (Figure 2B).

The islets from NCD-fed GPR92-KO mice exhibited a downregulation of the insulin transcription regulator, pancreatic and duodenal homeobox 1 (Pdx1), and an upregulation of MAF BZIP transcription factor B (MafB), an immature β cell marker (Supplemental Figure 2H). These augmented immature cells in the islets from NCD-fed GPR92-KO mice can also be attributed to immune cell infiltration, which is known to be an early sign of T1D/T2D (27). We next tested whether the insulin secretory ability of the islets of WT and KO mice under different diets was affected by IMs.

The islets of NCD-fed GPR92-KO mice exhibited reduced insulin secretion, which was restored by the depletion of IMs by clodronate treatment (Figure 2H). Upon HFD feeding, the islets from both WT and KO mice exhibited lower glucose-stimulated insulin secretion (GSIS) compared with the islets of NCD-fed WT mice, which is consistent with the finding that islets extracted from HFD mice have a time-dependent expansion of IMs and, consequently, impaired in vitro GSIS (28). The diminished insulin secretion in the islets from HFD-fed mice was also rescued by the depletion of macrophages (Figure 2H). However, the effect of clodronate was even greater in the islets from HFD-fed GPR92 KO mice (Figure 2H).

### GPR92 deficiency triggered proinflammatory responses and increased M1-like macrophage expansion in islets.

Since a marked expansion of immune cells and increased production of proinflammatory cytokines and chemokines leading to the abrogation of β cell functions are common features of diet-induced obesity and T2D (7, 8, 11), we next evaluated the effects of GPR92 deficiency on the inflammatory features of IMs.

**Figure 3:**
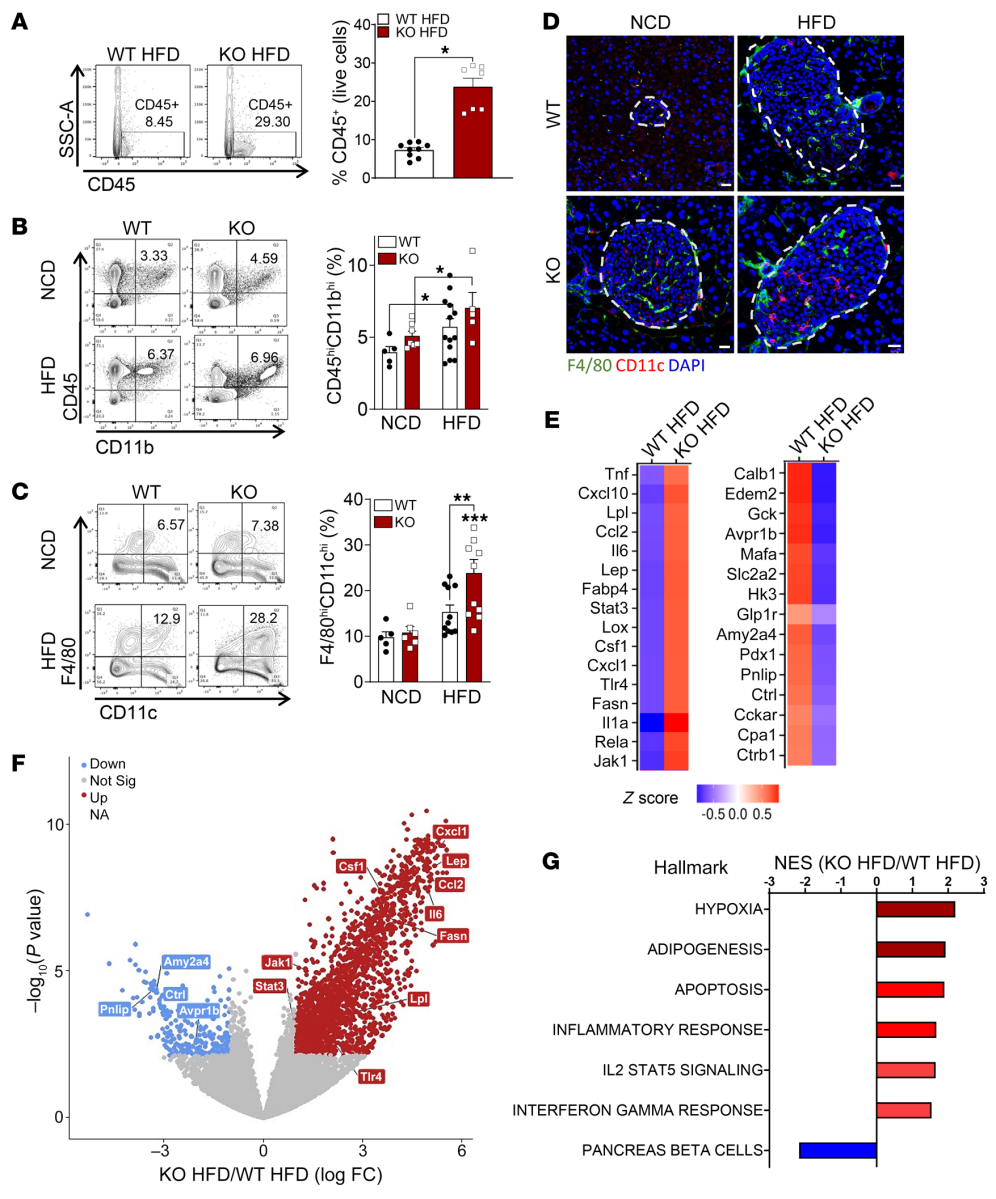
GPR92 deficiency triggers proinflammatory responses and increases the expansion of M1-like IMs. (**A**) Flow cytometry analysis of CD45^+^ cells in the islets from WT or KO mice on a HFD, *n =* 6–4/group. (**B**) CD45 and CD11b double-positive cells (Q4) in the islets from WT and KO mice on a NCD or a HFD, *n =* 3–4/group. (**C**) F4/80 and CD11c double-positive cells (Q4) in the islets from WT and KO mice on a NCD or a HFD, *n =* 3–5/group. (**D**) Immunofluorescence of the pancreas. Scale bars: 20 μm. (**A**–**D**) Data and images are representative of at least 3 independent experiments. All data are expressed as mean ± SEM. ***P < 0.001, **P < 0.01, *P < 0.05 by 2-way ANOVA with Bonferroni’s post hoc. (**E**) Top 15 most-relevant, upregulated (red) and downregulated (blue) genes in the islet from WT versus KO mice on a HFD. Mean values from *n =* 4–5/group. (**F**) Volcano plot of the fold change (*x* axis) versus adjusted (adj.) *P* value (*y* axis) of the transcriptomes between the islets from WT and KO mice on a HFD. Genes highlighted in red or blue are based on the thresholds of log_2_ fold change > 1 and adj. *P* < 0.05. (**G**) Pathways of the islets from WT versus KO mice on a HFD that were either activated (red) or repressed (blue) by lack of GPR92. Normalized enrichment score (NES) is represented in log_10_(*P*).

We observed a high abundance of leukocytes (CD45; Figure 3A and Supplemental Figure 3A), antigen-presenting cells (Figure 3B and Supplemental Figure 3B), and M1-like macrophages (F4/80 and CD11c; Figure 3, C and D) in the islets of HFD-fed GPR92 KO mice. Similarly, the RNA-Seq analysis of isolated islets from GPR92 KO mice on HFD revealed that several genes were upregulated — predominantly related to inflammation, such as *Tnfa*, *Ccl2*, *Il6*, and *Tlr4*, compared with WT mice on a HFD (Figure 3, E and F). In contrast, the genes downregulated in the islets of KO mice on a HFD were mostly associated with β cell function related to glucose uptake and metabolism (*Gck*, *Slc2a2*, and *Hk3*; Figure 3E) or β cell maturity (*MafA* and *Pdx1*; Figure 3F). The in-depth analysis of the pathways revealed that the upregulated genes in KO islets of HFD-fed mice were associated with hypoxia, adipogenesis, apoptosis, and inflammatory responses and signaling, whereas the downregulated genes were primarily related to pancreatic β cell function (Figure 3G). The higher inflammatory response and immune cell recruitment observed in the islets of KO mice on a HFD were also verified using qPCR. The islets from HFD-fed GPR92 KO mice exhibited a significant upregulation of various macrophage markers (*Csf1r*, *F4/80*, *Cd11b*, *Cd11c*, *Cd86*, *Cd206*, and *Cd36*; Supplemental Figure 3C) and dendritic cell markers (*Cd11c* and *Clec9a*; Supplemental Figure 3D) compared with islets from HFD-fed WT mice. T cell markers (*Ifng*, *Cd4*, and *Cd8a*; Supplemental Figure 3E) were upregulated in the islets from HFD-fed KO mice. Importantly, the islets from HFD-fed KO mice exhibited a 4-fold increase in the expression of *Ifng*, *Cd4*, and *Cd8a* (Supplemental Figure 3E) compared with the islets of NCD-fed KO mice. In addition to the increased expression of immune cell markers, the islets from HFD-fed KO mice exhibited an upregulation of several M1-like, proinflammatory genes, such as *Inos*, *Mcp1*, *Il6*, *Tnfa*, and *Il1b* (Supplemental Figure 3F). Supplemental Figure 3G demonstrates that the islet isolation was successful as exocrine enzymes were almost undetectable in the islets.

### GPR92 activation promoted antiinflammatory responses in islets and improved β cell insulin secretion.

Next, we tested whether GPR92 activation could promote the antiinflammatory response and subsequently ameliorate the β cell function. GPR92 is also known as an LPA-receptor; however, our published data (18) and data herein (Supplemental Figure 4A) established FPP as a strong GPR92 agonist. Since GPR92 is a G_q/11_-coupled receptor, it stimulates both PKC and MAP kinase, and both of these biologic effects are detected by serum response element-driven luciferase reporter system (SRE-luc) (29). HEK293 cells transfected with GPR92 and SRE-luc and further treated with various concentrations of FPP and LPAs exhibited a higher luciferase intensity at lower doses of FPP than at LPA 16:0 and LPA 18:0 (Supplemental Figure 4A). Similarly, FPP, but not LPA16:0, induced antiinflammatory effects, significantly reducing the intensity of NF-κB-luc activity in HEK293-TLR4 cells transfected with GPR92 and subsequent treatment with LPS (Supplemental Figure 4B).

**Figure 4:**
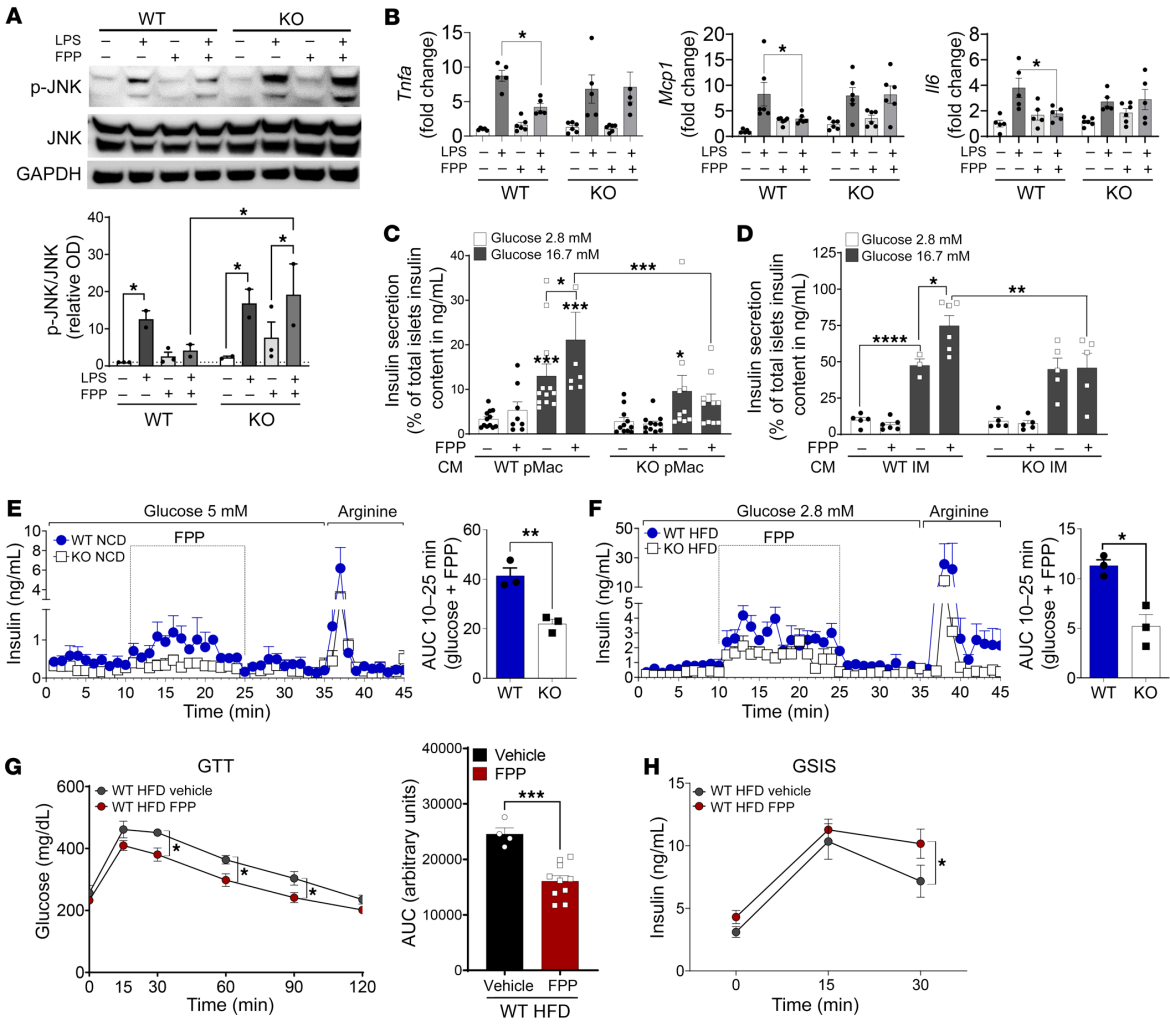
GPR92 activation promotes antiinflammatory responses and improves β cell function. (**A**) Peritoneal Macrophages (pMacs) from WT and KO mice cultured in vitro with or without FPP (10 μM) for 1 hour and then treated with LPS (1 ng/mL) for 15 minutes to detect JNK phosphorylation. The top panel is a representative image of 3 independent experiments, and the bar graph (bottom panel) shows fold induction over basal after normalization for total JNK. (**B**) Gene expression of proinflammatory mediators, *Tnfa*, *Mcp1*, and *Il6* in the islets from WT and KO mice cultured in vitro with (+) or without (–) FPP (100 μM) for 24 hours and then treated with LPS (+) (1 ng/mL) or PBS (–) for 2 hours, *n =* 5–6/group. (**C** and **D**) GSIS of WT islets cultured in vitro with conditioned medium (CM) obtained from WT or KO (**C**) pMacs or (**D**) IMs previously incubated with or without FPP (100 μM) for 24 hours. The islets were cultured with CM diluted to 1:5 to reﬂect the macrophage/β cell ratio in HFD-mice islets, (**C**) *n =* 9–10/group, (**D**) *n =* 4–6/group. The insulin secreted in the GSIS (ng/mL) was normalized by the islets total insulin (ng/mL) and then multiplied by 100. (**E** and **F**) Insulin secretion (ng/mL/min) of perfused pancreas from WT versus KO on a (**E**) NCD or (**F**) HFD. Pancreas was perfused with a basal glucose concentration (2.8 or 5 mmol/L) for 35 minutes, FPP (20 μM) was added to the perfusate during 10–25 minutes, and arginine (10 μM) from 35–45 minutes, *n =* 3/group. The bar graph shows the incremental AUC values of insulin secretion during FPP infusion. (**G**) GTT in WT mice on a HFD treated with FPP (0.1 mg/kg, i.p.) or saline (vehicle) for 1 week, and respective AUC, *n =* 4–11/group. (**H**) GSIS in WT mice on HFD treated with FPP or saline (vehicle) for 1 week, *n =* 4–11/group. All data are expressed as mean ± SEM. ****P < 0.0001, ***P < 0.001, **P < 0.01, *P < 0.05 by 2-way ANOVA with Bonferroni’s post hoc.

We validated this antiinflammatory effect driven by GPR92 activation in primary macrophages isolated from NCD-fed WT and GPR92-KO mice. We found that FPP pretreatment markedly inhibited LPS-induced phosphorylation of c-Jun N-terminal kinase (JNK) in WT peritoneal macrophages (pMacs), whereas GPR92-KO pMacs did not respond to GPR92 agonist or FPP pretreatment (Figure 4A). Similarly, FPP pretreatment reduced the expression of proinflammatory factors *Tnfa*, *Mcp1*, and *Il6*, induced by LPS treatment in cultured islets from WT mice. However, FPP failed to modulate the LPS-induced inflammation in cultured islets from GPR92 KO mice (Figure 4B). Together, these results demonstrate that the GPR92 agonist FPP promoted antiinflammatory effects on macrophages via GPR92 activation.

To examine whether the GPR92-mediated antiinflammatory effects on macrophages could influence the functions of β cells, we treated WT and KO pMacs with FPP. After 24 hours of treatment, we added the obtained conditioned medium (CM) from pMacs to WT islets. The ratio of CM to islets was 1 to 5, similar to the macrophage to β cell ratio observed in HFD mouse islets. As seen in Figure 4C, basal insulin secretion at a low-glucose concentration was unaltered by the addition of FPP. However, the GSIS was significantly increased by the addition of CM from FPP-treated WT pMacs after stimulation with high levels of glucose, while there is only a slight increased with the addition of CM from KO pMacs. Interestingly, the FPP treatment of KO pMacs exhibited no further effects (Figure 4C). We performed similar experiments with presorted IMs (F4/80^+^CD11b^+^ cells) from WT and GPR92-KO mice. In the presence of high glucose levels, the CM from WT IMs treated with FPP increased insulin secretion in the islets similar to the addition of CM from WT pMACs with FPP treatment, whereas the CM from FPP-treated GPR92-KO IMs did not further enhance the release of insulin (Figure 4D).

To gain insight into how FPP would modify macrophage function to influence β cell activity, we performed a proteomic analysis of the CM obtained from pMacs treated with FPP (Supplemental Figure 4C). The gene set enrichment analysis revealed that the CM from KO pMacs exhibited an enrichment of proteins associated with the adaptive immune response, such as IL-12, JAK/STAT, and MHCII pathways (Supplemental Figure 4D), whereas the CM from WT pMacs treated with FPP showed an enrichment of proteins predominantly associated with cellular communication and calcium binding (Supplemental Figure 4E).

Besides its antiinflammatory role, FPP treatment increased the expression of GPR92 (Supplemental Figure 4F) and insulin (Supplemental Figure 4G) in WT islets. Similarly, the direct perfusion of FPP into the pancreas of NCD-fed (Figure 4E) and HFD-fed WT mice (Figure 4F) increased insulin secretion. However, insulin secretion was enhanced by FPP only in the presence of GPR92. Hence, regardless of the diet, FPP did not promote insulin secretion in GPR92-KO mice (Figure 4, E and F), demonstrating an effect solely dependent on GPR92.

Additionally, we determined that systemic administration of FPP improved glucose intolerance in HFD-induced obese WT mice (Figure 4G), an effect that is mediated through stimulation of insulin secretion (Figure 4H). Similar to the direct pancreatic infusion, systemic treatment of FPP improved the glucose clearance and increased the insulin secretion only in the WT mice; FPP treatment did not improve the GTT (Supplemental Figure 4H) or GSIS (Supplemental Figure 4I) in GPR92 KO mice on a HFD.

## Discussion

In this study, we present an assessment of immunological and metabolic functions of what we believe to be a novel GPCR, namely, GPR92. We showed that GPR92 is highly expressed in IM populations and its expression is closely related to diet-induced obesity. In GPR92 deficiency, the islets from obese mice exhibit an increased expression of inflammation-associated mediators and enhanced M1-like macrophages. These IMs lead to β cell dysfunctions and impair the insulin secretion in HFD-fed GPR92-KO mice, an effect that is eliminated when these immune cells are depleted. Additionally, GPR92 activation by FPP modulates the macrophage inflammatory response, thereby restoring the insulin secretion by β cells. Our results clearly indicate that GPR92 deficiency increases the activation of IMs and leads to a proinflammatory phenotype. Moreover, our findings suggest that enhanced islet inflammation contributes to the abrogation of insulin secretion observed in islets of GPR92-deficient mice upon HFD feeding.

The pathogenesis of islet inflammation in T2D is a complex process, involving immune cell infiltration, cytokine production, β cell apoptosis, amyloid deposition, and islet fibrosis (1, 9). Our findings show that GPR92 colocalizes with islet immune cells, especially with macrophages (F4/80^+^ cells), whose population is substantially increased during obesity (8, 10, 11). Increased macrophage infiltration into pancreatic islets has been observed in patients with T2D (14) and in rodent models of insulin resistance (8, 11). Obesity predisposes the monocytes of HFD-fed mice to a proinflammatory phenotype upon their differentiation into macrophages (8). In the present study, we established a precise link between IMs and GPR92, as indicated by the increased expression of this GPCR by HFD, which is restored by the depletion of IMs. Thus, we hypothesized that obese GPR92 KO mice would exhibit weakened metabolic activity. Interestingly, GPR92 deficiency in IMs not only impaired insulin secretion but also enhanced the proliferation of immature β cells. Our results, therefore, suggest that immune cell migration and activation toward a proinflammatory phenotype is stimulated during the development of obesity and T2D (3, 7, 8), and this intensifies even more in the absence of GPR92.

Previous studies have established the role of IMs in the compensatory proliferation of β cells and obesity (8, 30, 31). Adaptive expansion of β cell mass has been observed in the prediabetic stage in rodent models of obesity (31–34). Furthermore, it has been shown that glucose (34, 35), insulin (36), and hepatocyte growth factor (33) promote β cell replication in obese mice. However, the underlying molecular mechanisms contributing to this phenotype and regulating this process remain obscure. Although various factors could directly affect β cells (34), IMs have also been known to play a central role in β cell neogenesis (8, 28) by secreting diverse soluble factors such as cytokines and chemokines. These immunomodulatory factors mediate the crosstalk between IMs and β cells (37, 38). In fact, our study provides evidence that the absence of GPR92 in IMs induces a more augmented inflammatory state, enhancing the proliferative capacity of β cells and directly abolishing the function of β cells.

In this study, we showed that GPR92-KO IMs severely diminish insulin secretion from islets, as supported by the reduced GSIS in the islets of NCD-fed GPR92-KO mice. However, a more striking observation is that the depletion of IMs had a substantial effect that led to an increase in GSIS in KO islets, independent of diet. With this approach, we believe that we have established the role of IMs in reducing the secretory function of β cells. Moreover, we observed that GPR92 deficiency can further exacerbate this process.

Other studies have shown that cytokine secretion affects β cell GSIS (1, 7–9). Interestingly, our findings showed that the absence of GPR92 triggers a remarkable inflammatory response in the islets of HFD-fed mice. The population of M1-like macrophages in the islets of HFD-fed GPR92-KO mice increased, and in addition, GPR92-deficient islets exhibited increased transcript-level expression of inflammation-associated genes, with overexpression of several cytokines, such as *Tnfa*, *Il6*, and *Il1*, and chemokines, such as *Ccl2* or *Mcp1*.

M1-like macrophages can secrete — and consequently increase the levels of — proinflammatory mediators in the extracellular environment of islets, thereby increasing the replication, infiltration, and activation of immune cells (39, 40). M1-like macrophages can also disrupt β cell functions, causing a substantial decrease in insulin secretion (8, 28). Therefore, we performed a proteomic analysis of CM obtained from pMacs to identify the potential factors that could impair the β cell functions in GPR92-deficient macrophages. Proteomic analysis demonstrated enhanced levels of many proinflammatory mediators. However, proteins related to MHC II antigen-presenting response and IL-12 signaling were markedly enriched in the CM from macrophages of HFD-fed GPR92-KO mice. Overexpression of MHC II antigen-presenting response–related genes in macrophages stimulates their engulfment function (41, 42); IL-12 promotes cell-mediated immunity by influencing the Th1 response, stimulating IFN-γ production and regulating the formation and release of early phagosomes (41, 43). Together, these findings suggest that GPR92-KO macrophages in the obese state can engulf intact β cell insulin-containing secretory vesicles (to a greater degree), thereby substantially inhibiting the insulin secretion in HFD-fed GPR92 KO mice. Other studies described a similar activity of the macrophages (8, 44). Thus, it is evident that the activity and the higher levels of proinﬂammatory cytokines secreted by GPR92 KO macrophages are responsible for the reduced insulin secretion.

With respect to the question of whether GPR92 activation could directly regulate immune cells, we hypothesized that GPR92 stimulation promotes antiinflammatory effects that would improve β cell functions. Our data on FPP, the stronger agonist of GPR92, validates this. FPP promotes the antiinflammatory effects in HEK293-TLR4 cells, WT macrophages, and islets, while simultaneously increasing insulin secretion. However, FPP was not able to promote the antiinflammatory responses and consequently improve GSIS in the islets of GPR92-KO mice. Hence, these findings suggest that the antiinflammatory action of FPP is exclusively exerted through GPR92. FPP interaction with GPR92 is known to activate β arrestin-2 (45), whose internalization inhibits the TAB1/TAK1 complex and abrogates downstream signaling to the IKKβ/NFκB and JNK1 pathways in another GPCR-mediated antiinflammatory mechanism (46). Therefore, FPP-driven antiinflammatory effects may be associated with the internalization of the GPR92/β arrestin-2 complex which will require future studies.

Our proteomic analysis showed an enrichment of proteins associated with cation transmembrane transport, focal adhesion, cellular communication, and calcium binding in the CM from WT macrophages treated with FPP. These GPCR-related key functions of proteins were substantially mitigated in the CM of GPR92-KO macrophages. Thus, one can speculate that in the absence of GPR92, FPP uptake is diverted to the mevalonate pathway (a cholesterol biosynthetic pathway), where it can be driven to several fates, or even be converted into a variety of lipid anchors, such as small GTP-binding proteins like Cdc42 (47), which mediates the activation of inflammatory pathways (41). However, further studies are warranted to conclusively establish this hypothesis.

Our findings strongly suggest that GPR92 regulates the function of pancreatic islets by modulating the IM inflammatory response. While the purpose of our current study is not to develop novel antidiabetic drugs, GPR92 in IMs could potentially be targeted to transactivate β cell function as a proficient therapeutic strategy to avert the damage caused by islet inflammation — which is caused by diet-induced obesity — and to regulate β cell-mediated insulin secretion in patients with T2D. In a future study, it would be worth determining whether the systemic treatment of FPP can be as potent as antidiabetic effects versus the current standard of care (e.g., metformin, GLP1R agonists, and/or SGLT2 inhibitors) or that FPP acts synergistically with these compounds when given chronically in a proper preclinical model of T2D.

Altogether, our findings demonstrate that GPR92, a GPCR expressed in IMs and highly modulated by a HFD, controls the chronic inflammatory pathways that drive the pathogenesis of obesity-related diabetes. GPR92-KO mice exhibit enhanced islet inflammation and severely diminished insulin secretion. However, GPR92 agonist, FPP, markedly reduced the inflammation in macrophages. Moreover, IMs treated with FPP positively modulated β cell GSIS. Therefore, our study establishes what we believe to be a novel endocrine role of GPR92 in islet-resident macrophages as it controls islet inflammation and β cell dysfunction. It also proposes GPR92 as a potentially novel therapeutic target to improve the clinical outcomes of T2D.

## Methods

### Animal care and use.

GPR92 (LPAR5)-KO mice were gifted by Jerrold Chun (Scripps Research Institute, La Jolla, California, USA) (20) and housed at a specific pathogen-free facility at UT Southwestern Medical Center. Male C57BL/6 (WT) or GPR92-KO littermates, from 8 weeks of age, were fed a NCD (13.5% fat; LabDiet) or a HFD (60% fat; Research Diet) provided ad libitum for 16–20 weeks. For FPP (Sigma-Aldrich) treatment, WT and KO mice fed with a HFD for 18 weeks were subjected to daily peritoneal injections of FPP (0.1 mg/kg) or saline (vehicle) for 1–2 weeks. Mice received a fresh diet weekly, and food consumption and body weights were monitored.

### Systemic tests and treatments.

For glucose tolerance tests (GTTs) or GSISs, mice were fasted for 4 hours before the administration of glucose (1.5 g/kg body weight by peritoneal injection or oral gavage, for GTT and GSIS, respectively). At the indicated times, venous blood samples were collected in heparin-coated capillary tubes. Glucose levels were measured using an oxidase-peroxidase assay (Sigma-Aldrich). Insulin levels were measured by a commercial ELISA kit (CrystalChem) as per the manufacturer’s instructions. Differences in glucose plasma levels against baseline were used to calculate the AUC values.

### Immunofluorescence staining.

For immunofluorescence paraffin sections, the pancreas tissues extracted from WT or GPR92 KO mice were fixed in PBS-buffered 10% formalin for 24 hours, embedded in paraffin, and sectioned (5 μm). The sections were fixed afterward in xylene and ethanol and subjected to antigen retrieval (8) before immunostaining was performed. For frozen sections, the pancreas tissues were fixed in 4% paraformaldehyde (PFA) for 2 hours, overnight in a 30% sucrose solution, and frozen in OCT (ThermoFisher Scientific). Tissue cryo-sections (10 μm) were fixed in 4% PFA and blocked/permeabilized with 10% normal goat serum and 0.5% Triton X-100 in PBS for 1 hour. Slides were incubated at 4°C overnight with the following primary antibodies (0.5–1 μg/mL), anti-mouse LPAR5 (GPR92) (ABT114, Sigma-Aldrich), anti-mouse Insulin (ab7842, Abcam), anti-mouse Glucagon (ab10988, Abcam), anti-mouse F4/80 (ab6640, Abcam), anti-mouse CD11c (ab33483, Abcam), anti-mouse CD45 (11045182, Invitrogen), anti-mouse MHCII (107614, Biolegend). The sections were then incubated with fluorochrome-conjugated secondary antibodies at room temperature, including anti-guinea pig Alexa Fluor 405 (ab175678, Abcam), anti-guinea pig Alexa Fluor 488 (A-11073, Invitrogen), anti-hamster Alexa Fluor 546 (A21111, Invitrogen), anti-hamster Alexa Fluor 488 (A-21110, Invitrogen), anti-rabbit Alexa Fluor 488 (10698447, Invitrogen), anti-rabbit Alexa Fluor 546 (A-11035, Invitrogen), anti-mouse Alexa Fluor 488 (A-10667, Invitrogen), anti-mouse Alexa Fluor 546 (A-11003, Invitrogen), anti-rat Alexa Fluor 488 (A-11006, Invitrogen). Images were acquired using a confocal microscope (Zeiss LSM 880 with Airyscan) and processed with ImageJ (https://imagej.net/software/fiji/).

### RNA isolation and quantitative reverse-transcription PCR.

Total RNA isolation and quantitative PCR were performed as described previously (48). Gene expression levels were calculated after normalization to the standard housekeeping genes RPL19 and B2M using the ΔΔCT method (46) and expressed as relative mRNA levels or fold change (2^–ΔΔCT^) compared with those of the control group. Primer sequences are listed in Supplemental Table 1.

### RNA-Seq.

Sequencing libraries were prepared according to the NEBNext Ultra II RNA Library Prep Kit for Illumina (New England BioLabs Inc.). Islets in Lysis Buffer were used as the starting material. RNA was extracted using the RNAqueous-Micro Total RNA Isolation Kit (Invitrogen). RNA-Seq analysis was performed as described by Shan and colleagues (2020, ref. 49). Quality-filtered reads were aligned to the mouse reference genome GRCm38 (mm10) by HISAT2 version 2.0.1 using default settings. Aligned reads were quantified using featureCounts version 1.4.6 per gene ID against mouse Gencode version 20. Analysis of differential gene expression was done using DEseq2 version 1.6.3. The cut-off values of fold change and FDR used to screen differentially expressed genes were described earlier in the text.

### Islet isolation.

The islets were isolated as described previously (8). Briefly, collagenase XI solution at 0.5 mg/mL (Sigma-Aldrich) was injected into the pancreatic common bile duct. After excision, the pancreas tissue was digested at 37°C for 15 minutes, centrifuged at 340*g* for 2 minutes, and filtered using a 100 μm cell strainer. The islets were purified by a discontinuous density gradient centrifugation (800*g* for 20 minutes without the break) generated by 3 layers, Histopaque 1119 (Sigma-Aldrich), Histopaque 1077 (Sigma-Aldrich), and HBSS (Gibco) with 0.1% BSA. The islets were collected from the interface between the second and third layers.

Primary mouse islets isolated from both NCD and HFD WT mice, fed for 18–20 weeks, were used to evaluate the effects of macrophages on GSIS. To deplete islet macrophages, the primary islets were handpicked (30 islets per each replication) and incubated in DMEM with 10 mL of clodronate liposome at 7 mg/mL (FormuMax). In control groups, the mouse islets were treated with empty liposomes at 7 mg/mL (FormuMax). After 24 hours, the islets were washed and incubated in Krebs-Ringer buffer (Sigma-Aldrich) with 2.8 or 16.7 mmol/L glucose for 1 hour. Insulin concentrations in the supernatant were determined by Ultrasensitive insulin ELISA kits (CrystalChem).

### IM sorting.

Isolated islets were dissociated in enzyme-free dissociation buffer (Sigma-Aldrich) at 37°C for 10 minutes, washed, filtered twice on a 40 μm cell strainer, and centrifuged at 600*g* at 4°C for 5 minutes (50). Cell pellets were resuspended in blocking buffer (PBS with 2% FBS) containing anti-mouse CD16/CD32 Fc Block [1:200], Invitrogen). Primary antibodies including anti-mouse CD45 FITC, 11-0451-82, eBioscience; anti-mouse F4/80 PE-CF594, 565613, BD Biosciences; anti-mouse CD11b BB700 rat, 566417, BD Biosciences; anti-mouse CD11c BV421 Armenian Hamster, 565451, BD Biosciences, were added to the cells in blocking buffer and incubated for 30 minutes at 4°C in the dark. After incubation, F4/80^+^ and CD11c^+^ IMs were washed once with PBS with 2% FBS and then resuspended in PBS with 2% FBS for sorting. IMs were sorted for collection using a BD Biosciences FACS Aria cytometer (UTSW Flow Cytometry Core Facility). Flow cytometry plots were generated with FlowJo version 10.

### Isolation of primary pMacs.

We harvested primary macrophages from the peritoneal cavity of WT and GPR92-KO mice as described previously (25, 51). After harvesting and plating overnight, the macrophages were provided fresh serum-free medium (DMEM, high glucose, with glutamine) for 24 hours. The macrophages and the CM were harvested and diluted into fresh serum-free DMEM to match the 1:5 cell ratio for in vitro GSIS experiments.

### In vitro GSIS experiments.

The previously-isolated islets were incubated with the CM (1:5) from pMacs or IMs for 24 hours and subjected to GSIS with glucose doses of 2.8 mM and 16.7 mM for 1 hour, as described previously (8).

### Proteomics.

For proteomics assays, the CM from primary macrophages was concentrated into Amicon Ultra-15 Centrifugal Filter Unit (Millipore). A total of 30 μg of protein loaded into a SDS page gel was removed and submitted to the UT Southwestern Proteomics Core. Briefly, samples were digested overnight with trypsin (Pierce) following reduction and alkylation with DTT and iodoacetamide (Sigma–Aldrich). The samples then underwent solid-phase extraction cleanup with an Oasis HLB plate (Waters) and were subsequently dried and reconstituted. A total of 2 μL of these samples were injected onto a QExactive HF mass spectrometer (ThermoFisher Scientific) coupled to an Ultimate 3000 RSLC-Nano liquid chromatography system. Samples were injected onto a 75 μm inner diameter, 15 cm EasySpray column (ThermoFisher Scientific) and eluted with a gradient from 0% to 28%, over 90 minutes with a flow rate of 250 nL/minute. MS scans were acquired at 120,000 resolutions in the Orbitrap and up to 20 MS/MS spectra were obtained for each full spectrum acquired using higher-energy collisional dissociation for ions with charges from 2 to 8. Dynamic exclusion was set for 20 seconds after an ion was selected for fragmentation. Raw MS data files were analyzed using Proteome Discoverer v2.4 (ThermoFisher Scientific), with peptide identification performed using Sequest HT searching against the mouse protein database from UniProt (downloaded on March 12, 2020; http://www.uniprot.org/). The FDR cutoff was 1% for all peptides.

### Pancreas perfusion.

The pancreas was perfused through the celiac artery and the perfusate was collected from the portal vein following a modified procedure of previous protocols (52, 53). Briefly, after anesthetization, a heparin solution (1,000 units/mL) was slowly injected into the mouse via the circulating blood through the vena cava to prevent blood clotting. Following this procedure, the perfusion medium (4.4 mM KCl, 2.1 mM CaCl_2_, 1.5 mM KH_2_PO_4_, 29 mM NaHCO_3_, 116 mM NaCl, 1.2 mM MgSO_4_, 20 mM HEPES, 1% [w/v] of BSA [fatty acid free], and 3% [w/v] dextran T40) was delivered to the ligated pancreas through the celiac artery at 1 mL/minute using a circulation pump (MP-II, Harvard Apparatus) and an in-line heater (Warner Instrument) set at 37°C. This solution was freshly prepared, filtered (0.45 mm), and equilibrated for over 10 minutes with 95% oxygen and 5% CO_2_. After an equilibrium period of 10 minutes, the perfusates were collected at 1 minute intervals for 45 minutes. Pancreas was perfused with a basal glucose concentration (2.8 or 5 mmol/L) for 35 minutes; 20 μM FPP was added to the perfusate during minutes 10–25, and 10 μM arginine was added between minutes 35–45. Insulin was measured in the perfusate using an assay kit (Cisbio).

### Statistics.

The values presented are expressed as the mean ± SEM. The statistical significance of the differences among 2 groups was determined by a Student’s 2-tailed *t* test. The difference among various treatments was determined by 1-way ANOVA or 2-way ANOVA with the Bonferroni’s post hoc using GraphPad Prism 7.0 software. Differences were considered significant when *P* < 0.05.

### Study approval.

All procedures were approved by the IACUC at UT Southwestern Animal Resource Center.

## Author contributions

DYO and COS conceived the project and designed the studies. COS performed most of the experiments. COS, VAP, and XS performed the mouse experiments. LV, QZ, and MS performed RNA-Seq library prep and data analysis. TO analyzed proteomic database. VAP, COS and NJ performed flow cytometry and cell sorting experiments. SC performed the pancreas perfusion assay. and LMAB assisted in experiments. RKG reviewed the manuscript and contributed to discussions. DYO and COS analyzed, interpreted data, and cowrote this manuscript.

## Supplementary Material

Supplemental data

## Version 1 09/06/2022

In-Press Preview

## Version 2 11/01/2022

Electronic publication
