# Supplementary material for: GPR92 activation in islet macrophages controls β cell function in a diet-induced obesity model
Source: J Clin Invest. 2022 Nov 1;132(21):e160097. doi: 10.1172/JCI160097 (PMC9621135; doi:10.1172/JCI160097)
Supplement: Supplemental data [file jci-132-160097-s181.pdf]

SUPPLEMENTAL INFORMATION

GPR92 Activation in Islet Macrophages Controls β Cell Function in a Diet-Induced Obesity Model

Camila O. de Souza[1], Vivian A. Paschoal[1], Xuenan Sun[1], Lavanya Vishvanath[1,2], Qianbin Zhang[1], Mengle Shao[1,3], Toshiharu Onodera[1], Shiuwei Chen[1], Nolwenn Joffin[1], Lorena Arango[1], Rana K. Gupta[1,2], Da Young Oh[1]*

[1]Touchstone Diabetes Center, Department of Internal Medicine, University of Texas Southwestern Medical Center, Dallas, TX 75390, USA; [2]Division of Endocrinology, Department of Medicine, Duke Molecular Physiology Institute, Durham, NC 27701, USA; [3]The Center for Microbes, Development and Health, Institute Pasteur of Shanghai, Chinese Academy of Sciences, Shanghai, 200031, China

* To whom correspondence should be addressed.

Da Young Oh, PhD (dayoung.oh@utsouthwestern.edu)

Supplemental Table 1. Primers used for qPCR (order of appearance)

Gene Official Symbol (Alias)	Forward primer (5' → 3')	Reverse primer (5' → 3')
Gpr92 (Lpar5)	ACCTGGACATGATGTTTGCCA	GAGACCAGTCGCCAATACCA
Ptprc (Cd45)	GAGCAGACCCGAGATCCAC	GCAGCACTACCAGAAAAGGCA
Csf1r	TGTCATCGAGCCTAGTGGC	CGGGAGATTCAGGGTCCAAG
Adgre (F4/80)	CCCCAGTGTCCTTACAGAGTG	GTGCCCAGAGTGGATGTCT
Itgam (Cd11b)	ATGGACGCTGATGGCAATACC	TCCCCATTCACGTCTCCCA
Itgax (Cd11c)	ACGTCAGTACAAGGAGATGTTGGA	ATCCTATTGCAGAATGCTTCTTTACC
Fgf1	CCCTGACCGAGAGGTTCAAC	GTCCCTTGTCCCATCCACG
Tgfb1 (TGFβ1)	AGACCACATCAGCATTGAGTG	GGTGGCAACGAATGTAGCTGT
Gcgr (GCG-R)	TGCACTGCACCCGAAACTAC	CATCGCCAATCTTCTGGCTGT
Gcg	ACTCACAGGGCACATTCACC	CCAGTTGATGAAGTCCCTGG
Pdx1	CCCCAGTTTACAAGCTCGCT	CTCGGTTCCATTCGGGAAAGG
MafA	AGGAGGAGGTCATCCGACTG	CTTCTCGCTCTCCAGAATGTG
MafB	TTCGACCTTCTCAAGTTCGACG	TCGAGATGGGTCTTCGGTTCA
Cd86	TGTTTCCGTGGAGACGCAAG	TTGAGCCTTTGTAAATGGGCA
Mrc1 (Cd206)	CTCTGTTCAGCTATTGGGACGC	CGGAATTTCTGGGATTCAGCTTC
Cd36	AAGCTATTGCGACATGATT	GATCCGAACACAGCGTAGAT
Ciita (MHCII)	TGCGTGTGATGGATGTCCAG	CCAAAGGGGATAGTGGGTGTC
Clec9a	GAAGTGCCAATCCCCTAGCAA	CAGTCACTACCTGAATGGAGAGA
Ifng (IFNγ)	ACAGCAAGGCGAAAAAGGATG	TGGTGGACCACTCGGATGA
Cd4	TCCTAGCTGTCACTCAAGGGA	TCAGAGAACTTCCAGGTGAAGA
Cd8a	CCGTTGACCCGCTTTCTGT	CGGCGTCCATTTTCTTTGGAA
Csf1	ATGAGCAGGAGTATTGCCAAGG	TCCATTCCCAATCATGTGGCTA
Nos2 (Inos)	AATCTTGGAGCGAGTTGTGG	CAGGAAGTAGGTGAGGGCTTC
Ccl2 (Mcp1)	TTAAAAACCTGGATCGGAACCAA	GCATTAGCTTCAGATTTACGGGT
Il6	CCAGAGATACAAAGAAATGATGG	ACTCCAGAAGACCAGAGGAAAT
Tnfa (Tnfα)	GCCACCACGCTCTTCTGCCT	GGCTGATGGTGTGGGTGAGG
Il1b (Il1β)	AAATACCTGTGCCCTTGGGC	CTTGGCATCCACACTCTCCAG
Amylase	TCACACGGGTGATGTCAAGTT	GTCTGGGTTAATGCTCACTTCTT
Carboxy-peptidase A2	GGTGATCCCGAATGATGAAGAG	GAACTCGGACATGGACTGTCT
Pancreatic Elastase 1	GTGGACACAGTACCGAGGAC	CCAGTTGCTTCGGATGAGGG
Insulin	CACTTCCTACCCCTGCTGG	ACCACAAAGATGCTGTTTGACA

Figure S1. de Souza, *et al.* --- related to Figure 1

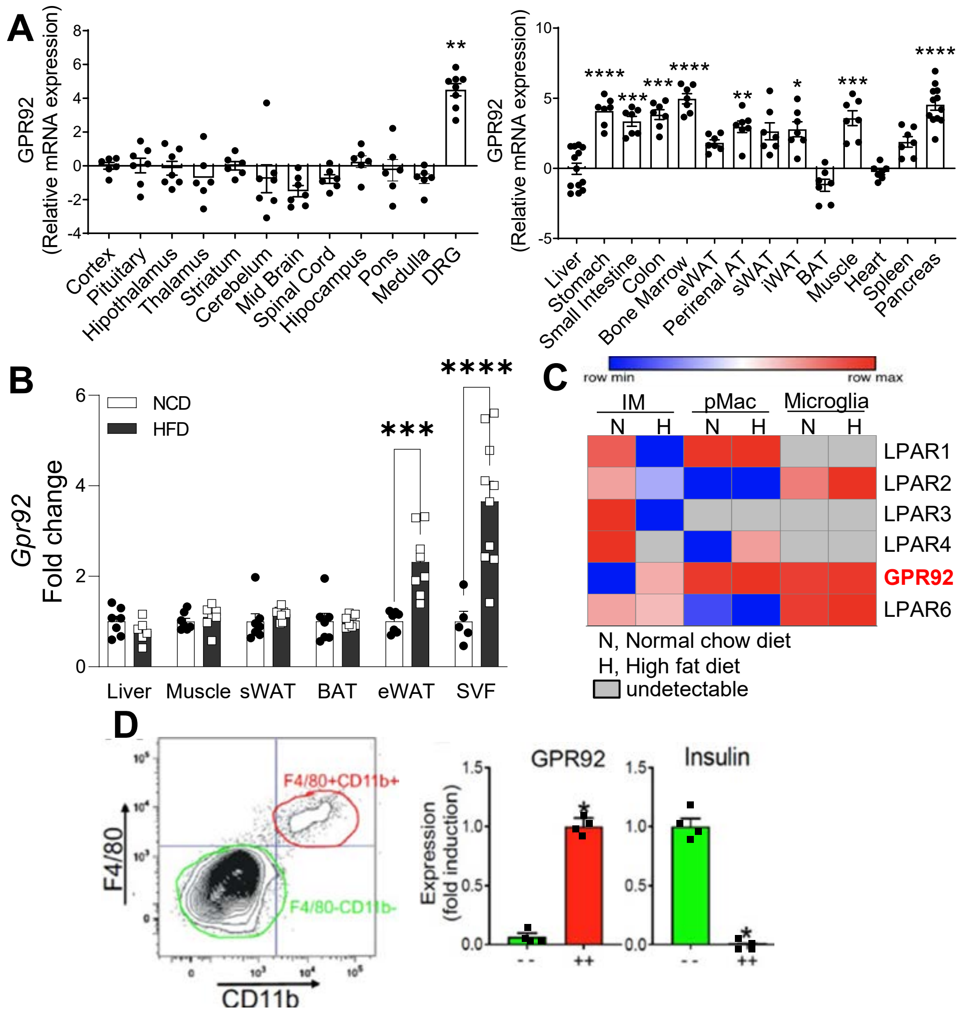

Figure S1 - Related to Figure 1. GPR92 is highly expressed in pancreas, and it is modulated by HFD in IM.
(A) *Gpr92* mRNA gene expression in nervous system tissues (left panel, relative changes to cortex) and peripheral organs (right panel, relative changes to liver), n = 6 to 8/group. **(B)** *Gpr92* mRNA gene in metabolic tissue of WT mice on NCD or HFD, n = 6 to 10/group. **(C)** Differential expression level of LPARs in IM, pMac and microglia from WT mice on NCD (N) *vs.* HFD (H) by RNA-seq data analysis (GSE133127), n = 4/group. **(D)** F4/80 and CD11c double-negative (- -, green) and double-positive cells (++, red) in the islets from WT-HFD and respective gene expression of GPR92 and Insulin, n = 3 to 5/group**.** See Table 1 for primer sequences. ΔΔCT normalized by *Rpl19* expression (A), Fold change normalized by *Rpl19* expression of Liver (B). Data are representative of at least two independent experiments. All data are expressed as means ± SEM. ****P < 0.0001, *** P < 0.001, ** P < 0.01, * P < 0.05 by one-way ANOVA with Bonferroni's post hoc (A) or by Student's t-test (B).

Figure S2. de Souza, *et al.* --- related to Figure 2.

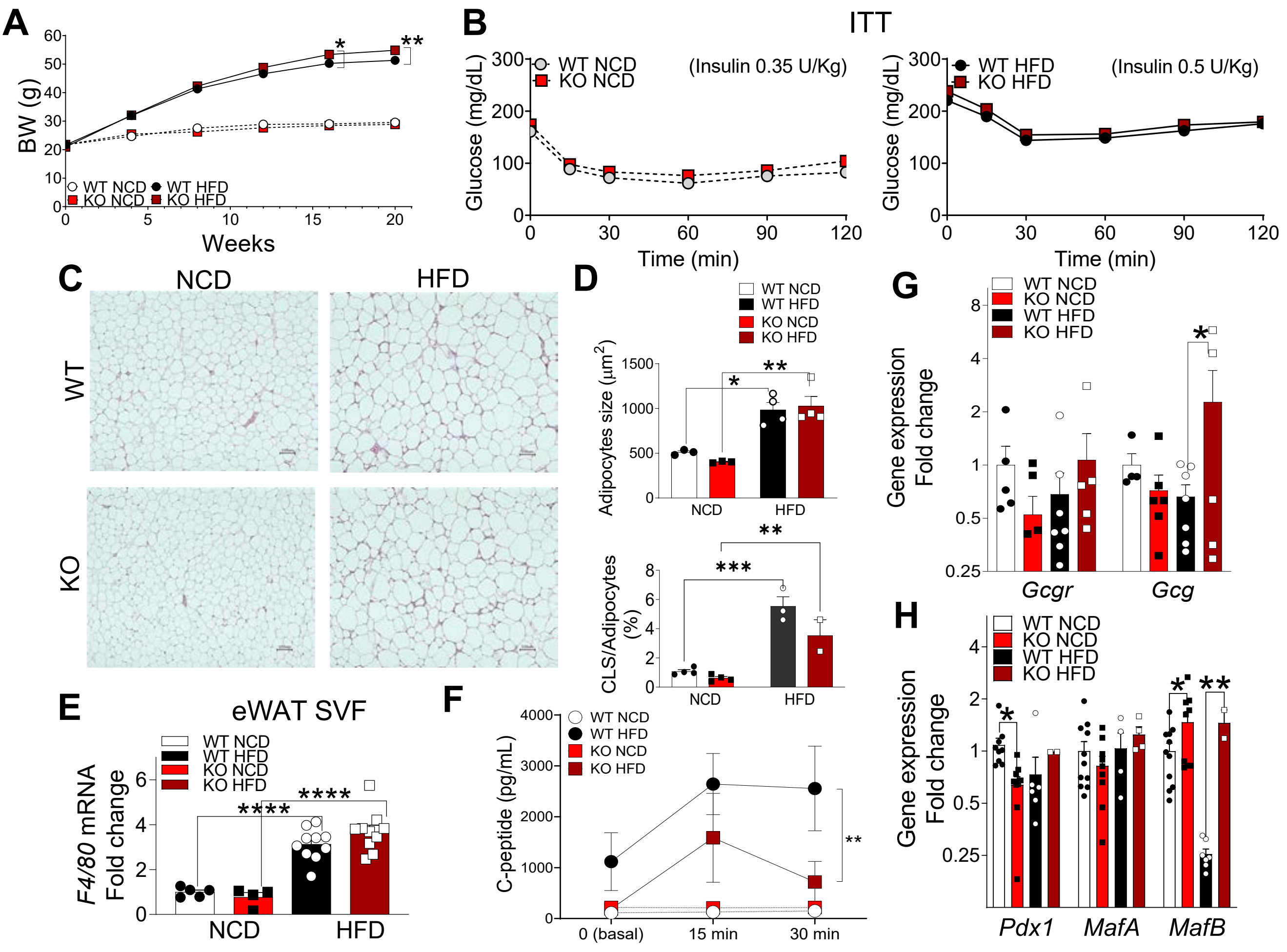

Figure S2 - Related to Figure 2. GPR92 deficiency does not enhance insulin resistance caused by HFD, but it disrupts islet function.
(A) Weekly body weight (BW) of WT or KO mice on NCD *vs.* HFD, n = 12 to 38/group. **(B)** ITT in WT *vs.* KO mice on NCD (left panel) or HFD (right panel), n = 8 to 25/group. **(C)** eWAT histology in WT *vs.* KO mice on NCD (left panels) or HFD (right panels); scale bars indicate 100 µm. Representative images are presented, n = 5 to 6/group. **(D)** Quantification of adipocytes size (µm^2) (top panel), and percentage of crown like structures (CLS) per adipocytes (bottom panel), n = 3 to 6/group. **(E)** *F4/80* mRNA gene expression in eWAT SVFs from WT *vs.* KO mice on NCD or HFD, n = 4 to 10/group. **(F)** Levels of C-peptide secreted on GSIS indicated in Fig. 2b, n = 12 to 23/group. **(G)** Gene expression of Glucagon-receptor (*Gcgr*), and Glucagon (*Gcg*) in islets, n = 5 to 7/group. **(H)** Gene expression of β-cell development markers, *Pdx1, MafA and MafB* in islets, n = 4 to 10/group. See Table 1 for primer sequences. Fold change normalized by *Rpl19* expression of WT-NCD. Data and images are representative of at least three independent experiments. All data are expressed as means ± SEM. ****P < 0.0001, ***P < 0.001, **P < 0.01, *P < 0.05 by two-way ANOVA with Bonferroni's post hoc.

Figure S3. de Souza, *et al.* --- related to Figure 3

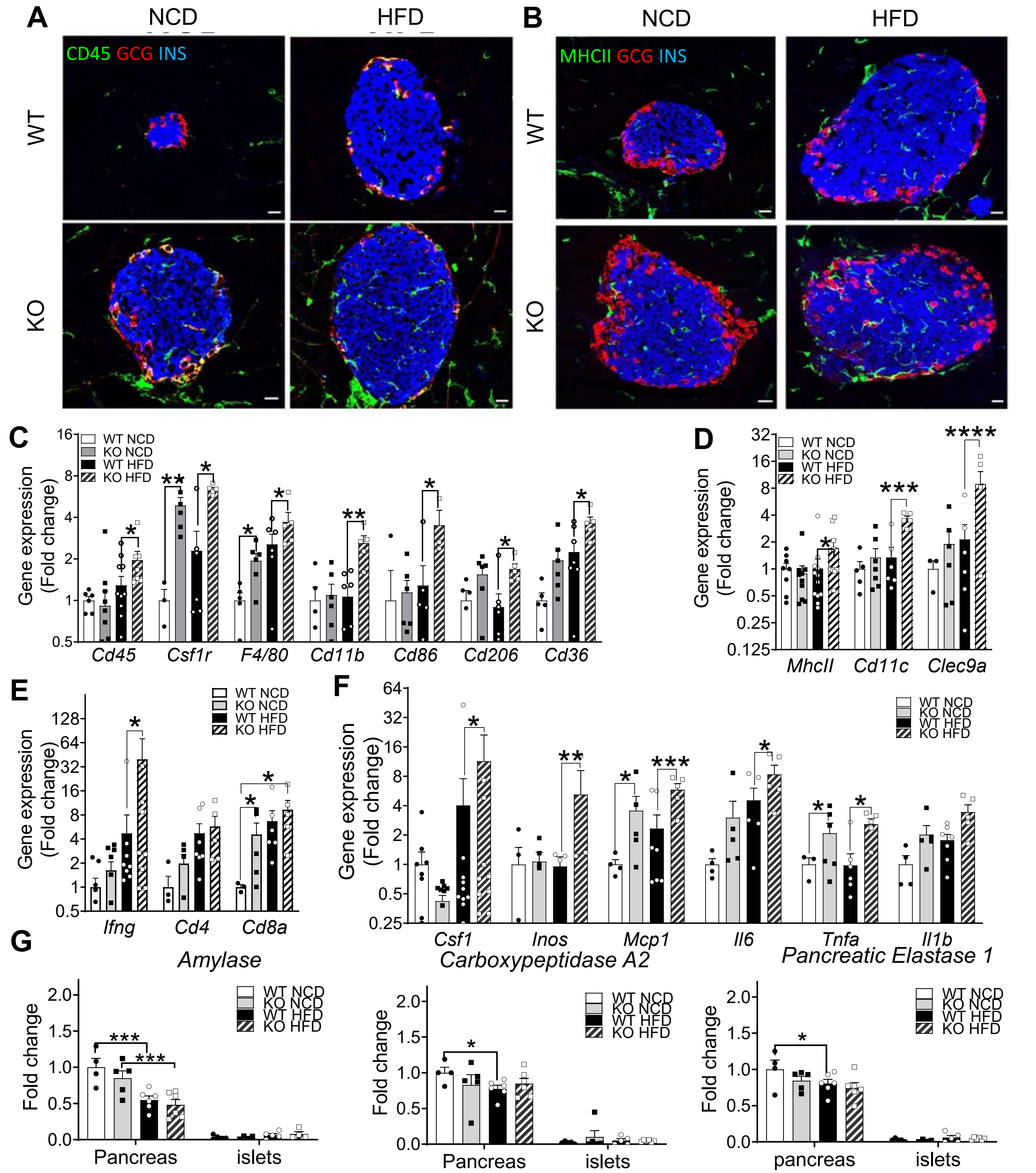

Figure S3 - Related to Fig. 3 Lack of GPR92 increases islet inflammation.
(A) Pancreas immunofluorescence shows increase of CD45⁺ cells in KO HFD mice; scale bars indicate 20 µm. Representative images are presented. n = 5 to 6/group. **(B)** Pancreas immunofluorescence shows higher MHCII cells in KO HFD mice; scale bars indicate 20 µm. Representative images are presented. n = 5 to 6/group. **(C)** Gene expression of *Cd45* and macrophage markers in islets, n = 3 to 6/group. **(D)** Gene expression of antigen presenting cells markers in islets, n = 3 to 6/group. **(E)** Gene expression of T-cell markers in islets, n = 3 to 6 per group. **(F)** Gene expression of pro-inflammatory mediators in islets, n = 3 to 5/group. **(G)** Gene expression of exocrine markers in pancreas and isolated islets, n = 4 to 6/group. See Table 1 for primers sequence. Fold change normalized by *Rpl19* expression of WT-NCD. Data and images are representative of at least two independent experiments. All data are expressed as means ± SEM. ****$P < 0.0001$, ***$P < 0.001$, **$P < 0.01$, *$P < 0.05$ by two-way ANOVA with Bonferroni's post hoc.

Figure S4. de Souza, *et al*. --- related to Figure 4

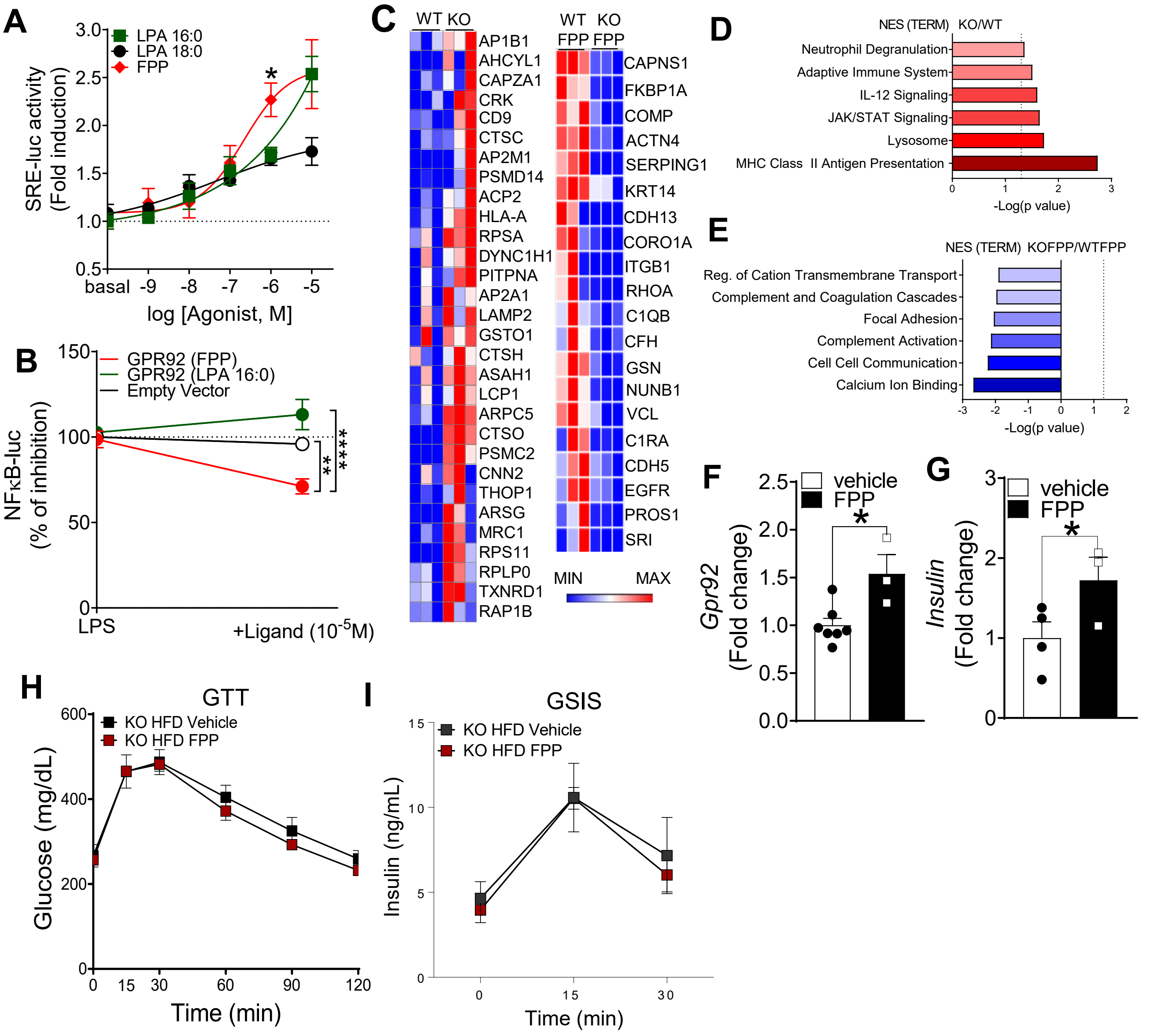

Figure S4 - Related to Figure 4. GPR92 stronger agonist, FPP, blocks NFκB pathway and increases GPR92 signaling in islets. **(A)** Luciferase activity in HEK293 cells transfected with GPR92 + SRE-Luc and treated with FPP, LPA16:0, and LPA18:0 for 6 h. Data are expressed as means ± SEM from triplication/each experiment of at least three or more independent experiments. **(B)** Inhibition of NFκB-luc activity (as % reduction) in TLR4-HEK293 cells transfected with GPR92 + NFκB-Luc reporter and then pre-treated with FPP or LPA16:0 for 6 h and subjected following treatment with LPS for 2 h. Data are expressed as means ± SEM from triplication/each experiment of at least three or more independent experiments. **(C)** Top 50 most relevant upregulated (red) and downregulated (blue) proteins in CM from WT *vs.* KO pMacs cultured under basal conditions (left panel), or treated with FPP (10 µM) for 24 h (right panel), n = 3/group. **(D)** Pathways activated (red) in CM from WT *vs.* KO ipMacs cultured under basal conditions. **(E)** Pathways deactivated (blue) in CM from WT *vs.* KO pMacs treated with FPP as indicated in C. **(F)** Gene expression of *Gpr92* in islets of WT mice treated with FPP (10 µM) for 24 h, n = 3 to 7/group. **(G)** Gene expression of *insulin* in islets of WT mice treated with FPP (10 µM) for 24 h, n = 3 to 4/group. **(H)** GTT in KO mice on HFD treated with FPP (0.1 mg/kg injected *ip*) or saline (vehicle) for 1 week, n = 3 to 6/group. **(I)** GSIS in KO mice on HFD treated with FPP or saline (vehicle) for 1 week, n = 3 to 6/group. See Table 1 for primers sequence. Fold change normalized by *Rpl19* expression of vehicle. All data are expressed as means ± SEM. Normalized enrichment score (NES) is represented in log10(P). ****$P < 0.0001$, **$P < 0.01$, *$P < 0.05$ by Student *t*-test.
